# Enhancement of Expression Level of Modified t-PA (TNKase) in *Leishmania tarentolae* by Induction System

**DOI:** 10.29252/.23.4.272

**Published:** 2019-07

**Authors:** Mohammad Mehdi Attarpour Yazdi, Nikky Tofighi, Taraneh Rajaee, Marzieh Ghahremanlou, Ahmad Adeli, Azam Bolhassani, Mohammad Azizi, Noushin Davoudi

**Affiliations:** 1 Department of Microbiology, Faculty of Medicine, Shahed University, Tehran, Iran; 2 Biotechnology Research Center, Pasteur Institute of Iran, Tehran, Iran; 3 Department of Hepatitis and AIDS, Pasteur Institute of Iran, Tehran, Iran

**Keywords:** *Leishmania tarentolae*, Tenecteplase, Tissue plasminogen activator

## Abstract

**Background:**

The expression of bio-therapeutic proteins in mammalian cells, such as CHO, attains high homogeneity related to post-translational modifications. Although CHO remains the most popular cell line for bestselling biotherapeutic proteins on the market, there are still drawbacks such as expensive culture media, long time line, and high drug cost. Recently, researches on a novel *Leishmania* protozoan system have confirmed that this low-level eukaryote could represent a competitive alternative to the mammalian cell lines.

**Methods:**

The full length of coding sequence of modified tPA TNKase (tenecteplase) was synthesized and cloned into an inducible expression vector of *L. tarentolae* T7-TR cells.

**Results:**

The expression of the construct was driven by a Tet-inducible promoter. A *Leishmania* secretory signal sequence was also added to the expression cassette to facilitate the release of the recombinant protein into the medium. The secretory recombinant protein was analyzed and confirmed by SDS-PAGE and Western blot analyses. The expression level of TNKase in this novel system of *L. tarentolae* was 810 IU/mL after induction, which means that the percentage of expression increases two times compared to previous models in *L. tarentolae*. The TNKase activity was comparable with Activase.

**Conclusion:**

Our results suggested that expressed TNK (modified tPA) is functionally compatible with Activase regarding their effect on fibrinolysis. Given the post-translational modification similarities between mammalian and *L. tarentolae*, it is speculated that this system is capable of producing complex proteins such as tPA similar to mammalian system, with easier manipulation and non-expensive method.

## INTRODUCTION

There are a variety of expression systems available for large-scale recombinant protein production. Despite various methodologies, there is no universally applicable expression system for producing recombinant protein. A proper expression system should be selected upon productivity level, bioactivity and chemical characteristics of produced protein and also the cost and safety of the system. As the need for quantity, purity, and quality improvement of bio-therapeutic products is growing, novel strategies for developing efficient eukaryotic cell systems become more mandatory[1].

Technical restrictions observed in using different systems have forced biotechnologists to look for new feasible and easy-handling systems like *E. coli*[2]. However, limitations of *E. coli* expression system causes to achieve only 15% active form of mammalian proteins[3]. *Saccharomyces cerevisiae* is the only eukaryote considered for high-throughput applications, but the nature of post-translational modifications in yeast cells is different from those in human cells. Very limited number of these expression systems can ensure the proper folding of complex human recombinant proteins[4]. Most eukaryotic expression systems based on insects, plants, and mammalians are slow for high throughput protein production, as the transcription of heterologous genes is mediated by a highly regulated RNA Polymerase II (Pol II). The feedback response between the overexpression of protein and the activity of RNA Pol II results in down-regulation of Pol II, leading to low expression yields[5]. In fact, RNA processing and RNA polymerase activity are coupled, which creates a regulatory step in the expression regulation of eukaryotic organisms to control protein overexpression[6]. It is believed that multiple steps such as signal peptide cleavage disulfide bond formation and glycosylation (N- and O-linked) are involved in post-translational modification of complex proteins (i.e., enzymes, co-enzymes, or antibodies) in bioactive form, which limits their expression[7]. These limitations were reasons to develop alternative expression systems capable of correcting post-translational modifications in recombinant proteins. Therefore, a new eukaryotic expression system with high level expression of recombinant proteins and less controlled polymerase activity and RNA processing is desirable. These objectives were achievable only in the members of the order Trypanosomatidae due to the natural uncoupling of transcription and RNA processing[5]. Among them, *Leishmania* *tarentolae*, a non-pathogenic parasite, has recently been investigated and employed as a potential eukaryotic expression host[8-10]. To date, several successful examples of using *L. tarentolae* as a protein expression system have been reported by our group and others[9-14].

Tenecteplase (TNKase, Genentech, USA) is a generic variant of tissue plasminogen activator (t-PA), created by recombinant DNA technology from a mammalian cell line. Like Activase, Tenecteplase is a 527-amino-acid glycoprotein with modifications at three sites of the t-PA (Activase) structure, i.e. three substitutions, at T103 to N, at N117 to Q, and at KHRR (296–299) to AAAA[15,16]. These substitutions have led to a longer half-life and higher fibrin specificity than t-PA and have shown slower inhibition by PA-1 in comparison with t-PA[17,18].

TNKase or Tenecteplase is an approved drug for treatments of acute myocardial infarction and stroke[19,20]. Herein, we chose TNKase as a protein model, which is a t-PA with longer half-life[17,20].

This study investigated the expression of TNKase, which is a large, complex, and highly glycosylated protein in an inducible *L. tarentolae* system. We also showed that the recombinant protein has biological activity as similar as mammalian-expressed drug (Activase).

## MATERIALS AND METHODS

### Cultivation and maintenance of *L. tarentolae*

*L. tarentolae* T7-TR strain (Cat.-No. EGE-1410, Jena Bioscience, Germany), were cultivated on brain heart infusion (BHI) medium (Merck, Germany), with the addition of 15 μg/L of hemin (Jena Bioscience), 50 IU/mL of penicillin, and 50 μg/mL of streptomycin (Jena Bioscience). As used *L. tarentolae* is T7-TR, the inducible host, two more antibiotics, hygromycin (50 µg/ml) and nourseothricin (50 µg/ml; Jena Bioscience), were added. Cells were cultivated in 50-ml ventilated flasks (Orange, USA) at 26 °C in two styles: static and agitated culture. The suspension culture of *L. tarentolae* was propagated by dilution ratio of 1:10 to 1:100 into a fresh medium when it reached the stationary growth phase. After dilution, the number of cells was typically 10^7^/ml.

### Construction of inducible-integrative expression vectors

**Fig. 1:**
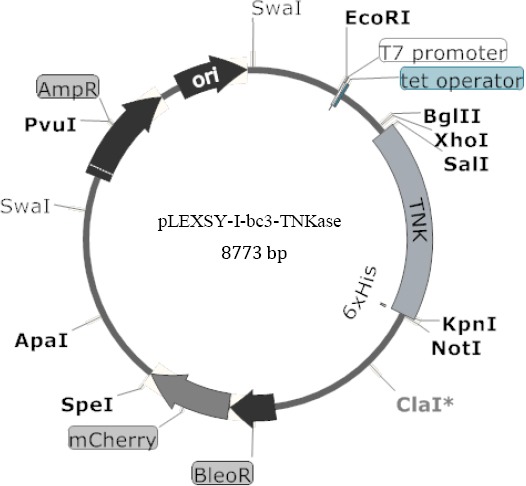
Map of the pLEXSY-I-blecherry3 vector, containing TNKase gene.

The DNA sequence of TNK was extracted from DrugBank database (www.drugbank.ca; accession no. DB00031) and was optimized upon Leishmania codon usage and synthesized commercially (Gencust Germany). The synthetic sequence was cloned into *Sal*I/*Kpn*I sites of an inducible LEXSY expression vector, pLEXSY-I-blecherry3 (Fig. 1). The cloning site is in front of *Leishmania mexicana* secretary acid phosphatase gene (*lmsap1*) signal peptide in vector and is in frame with the target protein, allowing the secretory expression of TNKase in Leishmania. The resulting plasmid was confirmed by PCR analysis and digestion with different enzymes.

### Transfection of *L. tarentolae* T7-TR

Initially, *L. tarentolae* T7-TR was grown as a static suspension in BHI broth as mentioned above. Afterwards, pLEXSY-I-blecherry3-TNKase plasmid was digested with *Swa*I restriction enzyme (Jena Bioscience), and the ~6830-bp fragment containing TNK gene was gel purified. For transfection, log- phase parasites were resuspended in 400 µl of ice-cold electroporation buffer (20 mM of HEPES, 137 mM of NaCl, 5 mM of KCl, 0.7 mM of Na_2_HPO_4_, and 6 mM glucose, pH 7.5), mixed with approximately 10 µg of the linearized developed expression cassette and then electroporated using Bio-Rad Gene Pulser (Bio-Rad, USA), at 500 µF and 450 V. The electroporated promastigotes were incubated in BHI broth medium at 26 °C for 24 h without any drug. The selection of single colonies was done by growth on solidified BHI medium containing 50 µg/ml of nourseothricin, 50 µg/ml of hygromycin, and 60 µg/ml of bleomycin (Jena Bioscience). After one week, transfected clones were appeared on solid media. Each clone was cultured and verified by PCR using TNKase specific primers (Table 1). To confirm the homologus recombination integration of the cassette containing TNKase into the *odc* locus of *L. tarentolae* genome in transfected strains, different diagnostic PCRs were performed. For this purpose, primer pairs, including one primer hybridizing within the expression cassette and one primer hybridizing to an *odc* sequence outside the developed plasmid, were applied. The information of primers is shown in Table 1. Genomic DNA from a 10-ml culture (OD ~2) was extracted by conventional phenol/chloroform extraction. The culture supernatant or precipitated *L. tarentolae* cells samples were subjected to SDS-PAGE analysis. The samples were mixed with SDS sample buffer (100 mM of Tris-HCl, pH 6.8, 20% [v/v] glycerol, 2% [w/v] SDS, 0.1% [w/v] Bromophenol blue, and 200 mM [v/v] of β-mercaptoethanol) and boiled for 3 minutes. Samples from both wild and transgenic *L. tarentolae* were separated on 12% (w/v) SDS-PAGE gel. For Western blot, the resolved proteins were transferred to the nitrocellulose blotting membrane (GE Healthcare, Germany) using a wet blotting system. The membrane was blocked two h at room temperature with 5% (w/v) skim milk in PBS containing 0.05% Tween-20 and then incubated for two h with 1/1,000 dilution of anti-tPA antibody (rabbit polyclonal antibody to tPA, Abcam, UK). After washing, the membrane was incubated in a 1/3,000 dilution of goat anti-rabbit IgG horseradish peroxidase (ab6721 Abcam) at room temperature for 2 h. Then target protein bands were developed by ECL kit (GE Healthcare, Norway).

**Table 1:** The primers used in this study

Primer	Sequence	Tm	Product (bp)
TNK Forward	5’-ATGGATGCAATGAAGAGAGG-3’	60	1600
TNK Reverse	5’-GGTCGCATGTTGTCACG-3’		
*3’odc*For (A708).	5’-GGATCCACCGCATGGCCAAGTTGACCAGTG-3’	60	2700
*odc* Rev (P1510)	5’-GTGCACCCATAGTAGAGGTGC-3’		

### Bioactivity assay

The plasminogen activation activity of expressed TNKase was assessed using agarose-fibrin plates according to the methods described previously with modifications[19,21]. The agarose fibrin plates were prepared as follows: 1.0 IU of thrombin, 0.75 g of plasminogen, and 30 IU of human fibrinogen were added to 12 ml of 1.0% agarose gel that had been dissolved in normal saline at 45–55 °C. The mixture was incubated at room temperature for 30 min. The sample was then loaded in wells made onto the plate, then incubated at 37 °C for 2 h. To determine the activity of the rTNKase, the standard tPA (Activase) was diluted and spotted onto the fibrin plate. Antibody against TPA was used for detecting the inhibition of t-PA lysis by anti-tPA action. The activity of the rTNKase was then measured based on the diameter of the clear zone on the fibrin plates.

### Amidolytic activity of rTNKase

Chromolize™ tPA kit (Biopool, USA) is a bio-functional immunosorbent assay designed for the quantitative determination of human tPA. The assay started by adding the samples to the micro test wells containing antibodies. The wells were washed with a mild detergent, and the sample was captured by antibodies on the micro test wells. Next, the substrate consisting of plasminogen and a plasmin sensitive chromogenic substrate was added. The amidolytic activity was calculated from the increase of absorbance at 405 nm.

## RESULTS

### TNKase sequence

The sequence of TNKase was optimized for codon usage of *L. tarentolae* (http://www.kazusa.or.jp), and then the optimized DNA sequence was synthesized. Two restriction sites, *Sal*I and *Kpn*I, were also added at the 5’ and 3’ends, respectively. The synthetic fragment was then cloned in pLEXSY-I-blecherry3. After confirmation of the obtained construct by digestion and PCR, the recombinant pLEXSY-I-blecherry3-TNKase vector (Fig. 1) was prepared in a large scale with high purity and linearized and transfected into parasites.

### ransfection of construct and clonal screening and induction

**Fig. 2:**
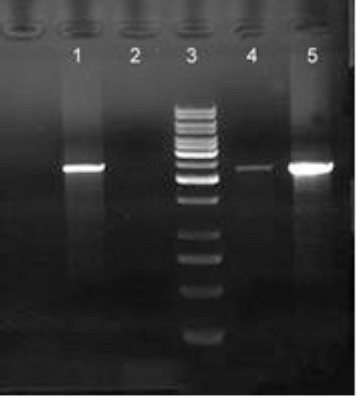
Confirmation of correct integration of TNKase gene in *odc* site by comparison between transfected clones and non-transfected clones. All correct integration showed a 2.7-kb PCR product with two specific primers A708 and P1510. Lane1, transfected *L. tarentolae* (clone 1); lane 2, non-transfected *L. tarentolae* clone; lane 3, DNA marker (1 kb); lane 4, transfected *L. tarentolae* (clone 2); lane 5, transfected *L. tarentolae* (clone 8).

The TNKase containing fragment (6400 bp) was cut out from pLEXSY-I-blecherry3-TNKase by *Swa*I digestion and transformed into *L. tarentolae* by electroporation. The transfectants were screened by plating on the BHI solid medium containing bleomycin, as a selection marker. Integration of TNKase gene in the genomic DNA of the recombinant parasites was confirmed by PCR. The expected ~1.6-kb band was observed in positive clones, indicating the existence of TNKase gene, and the 2.7-kb PCR product confirmed the integration of expression cassette into the *odc* locus of chromosome 12 of *L. tarentolae* (Fig. 2). For confirmatory PCR, primer pairs (Table 1), including one primer for annealing within the expression cassette as A708 and one primer hybridizing to a chromosomal *odc* sequence as P1510 (Jena Bioscience), were used. Two out of 20 colonies were selected for protein expression.

### Purification of soluble tPA protein

**Fig. 3:**
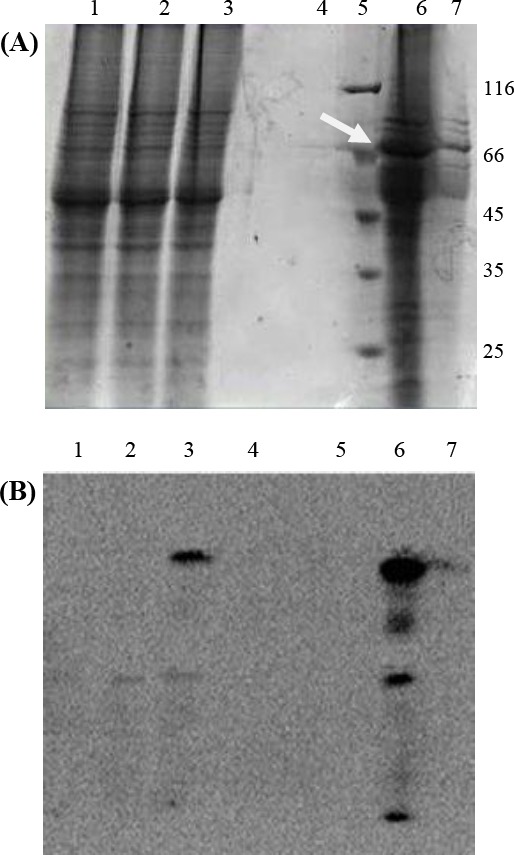
SDS-PAGE (A) and Western blot (B) analyses of *L. tarentolae* culture media expressing TNK. Orders of lanes are the same in both Figures. Lane 1, 30 μg of cell lysis of non-transfected *L. tarentolae;* lane 2, 30 μg of cell lysis of transfected *L. tarentolae* without induction at 72 h; lane 3, 30 μg of cell lysis of transfected *L. tarentolae* with induction at 72 h; lane 4, empty well; lane 5, protein size marker (116-14 kDa); lane 6, 30 μl of concentrated supernatant of transfected *L. tarentolae* induced for 72 h (68 kDa target band is indicated by arrow); lane 7, 30 μl of concentrated supernatant of non-induced transfected *L. tarentolae*

To verify the expression of TNKase, the cells were cultured in BHI in a shaker incubator under induction by 10 mg/ml tetracycline. The cells were harvested at 24, 48, and 72 h after induction. Despite the manufacturer’s suggestion, in which the best time of protein expression was 48 h after induction by tetracycline, TNKase expression was detectable in cell culture supernatant 24 h after induction and increased in time up to 72 h. SDS-PAGE analysis on supernatant of induced culture of transfected *L. tarentolae* confirmed the high level expression of TNKase after induction related to W/O induction samples (Fig. 3A). The expression was confirmed by Western blotting in cell extracts of transgenic parasites (Fig. 3B). The dominant band of ~68 kDa was observed in culture supernatant of recombinant parasites but not in the wild-type Leishmania culture (Fig. 3B). As secreted TNKase protein contained C-terminal polyhistidine Tag, the purification was done by affinity chromatography.

### Characterization and activity of the recombinant TNKase

**Fig. 4:**
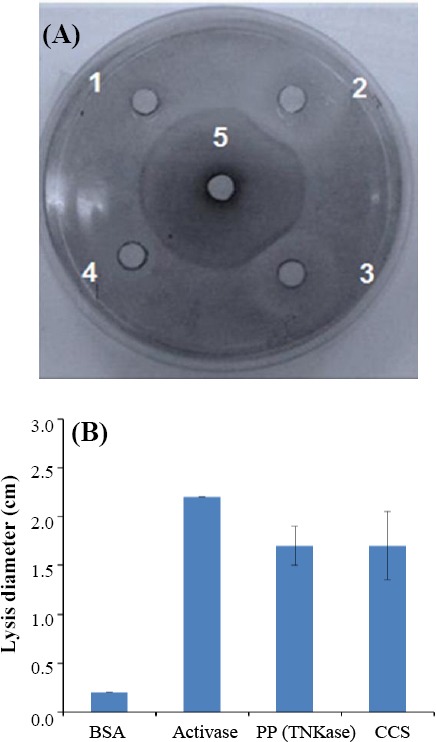
Lysis on a fibrin plate using Activase and expressed rTNKase. (A) Activase standard (positive control); BSA, negative control. The fibrinolytic activity of TNKase was measured and compared with Activase (positive control) and BSA (negative control). Well 1, BSA; well 2, Activase; well 3, purified protein (TNKase); well 4, cell culture soup; well 5, Ab against t-PA (the Ag/Ab reaction shown as dark circle around the dot). (B) the diameters of the lysis on the fibrin plate from three independent experiments. The bars indicate standard error of the mean. PP, purified protein; CCS, cell culture soup

For characterization of the recombinant TNKase, fibrin plate lysis assay was used. In order to assess whether the rTNKase was biologically active, plasminogen was used as substrate in the medium on the plate. Active TNKase was able to bind to plasminogen similar to tPA (Activase; Genentech) and to cleave it into plasmin, which degrades fibrin and results in a clear lysed zone on the fibrin/agar plate. In the center of plate, antibody against tPA (Abcam, Germany) was used. Upon the interaction of commercial antibody withthe expressed TNKase (no. 3) and standard tPA (Activase; no. 2), the immunopercipitation dark line, which is Ag/Ab complex, was developed. Migration of samples in other wells was inhibited by Ag/Ab complexes, i.e. transfected cell supernatant without purification (no. 4) with weak reaction related to Ab (no. 5) and BSA (no. 1) was used as the negative control which did not show any reaction (Fig. 4).

### Amidolytic activity test

**Fig. 5:**
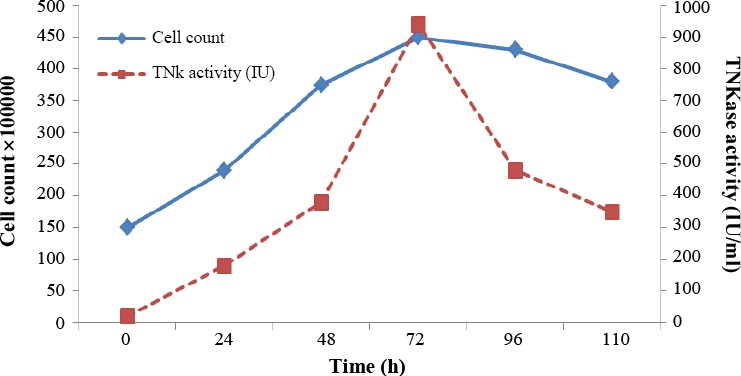
Amidolytic activity of secreted TNKase transfected *L. tarentolae* culture supernatant plotted against the average cell count during a five-day agitated culture. Expression level data represent means obtained from three experiments.

Supernatant of TNKase positive cells were used for the assessment of amidolytic activity. After Affinity purification procedure, for quantitative determination of TNKase biological activity, in supernatant of transfected *L. tarentolae*, a bio-functional immuno-sorbent assay was performed. Expression level of transfected cells was determined during five days (24 h to 110 h) post culture and with different amounts of tetracycline for induction in cultures using T flasks with starting cell density at 1.5 × 10^6^ cell/ml. As shown in Figure 5, the highest expression level was obtained on day four. The viability of cells reduced from 95% on day one to 70% on day four. Based on Biopool Chromolize t-PA assay kit, the highest expression level was detected as 810 U/ml on day three of culture in an agitated condition and 10 µg of tetracycline. Figure 5 shows the growth curve of transformed *L. tarentolae* in an agitated culture and the enzymatic activity of the rTNKase produced by transformed cells. The growth curves of transformed and non-transformed cells were almost identical, and therefore, only the average growth curve was plotted.

## DISCUSSION

The majority of recombinant proteins in clinical use are expressed in mammalian cells, which offer native glycosylation and folding of the target proteins. Due to incapability of prokaryotic systems in processing and secretion of complex recombinant proteins, the development of eukaryotic expression systems has proceeded in last two decades.

In eukaryotic systems such as mammalian cell, RNA Pol II is responsible for the transcription of genes in a manner of complex protein containing different transcription factors. Introduction of simple prokaryotic transcription machinery in a eukaryotic system is one of the strategies having advantage of higher system without the complex transcription regulation. Based on described strategy, a novel eukaryotic protein expression system has recently been developed in *L. tarentolae*[8].

In our previous report, the expression of full length rtPA was examined in two *L. tarentolae* cell lines using different constructs without induction[10,12,13]. The synthetic cDNA of tPA, with its own signal peptide, was used for expression in pFX1.4sat and pFX1.4hyg vectors. The expression level of rec-tPA in transfected *L. tarentolae* P10 was achieved up to 0.17 μg/ml equal to 70 IU/ml. Different levels of recombinant protein expression have also been reported from our and other labs, using different *L. tarentolae* expression systems[9-12,17,18].

Expression of truncated rtPA containing only the kringle-2 and serine protease domains using pF4splmsapx1.4 hyg vector has shown a secreted biologically active enzyme with the yield of 931 IU/mL[13]. It has also been indicated that the gene dosage affects the expression level and stability of full length t-PA in *L. tarentolae* progeny[12].

Based on our previous experiences, in an attempt, we examined the expression of TNKase (modified tPA) in *L. tarentolae* under the control of inducible T7 promoter. As it is believed that translational selection is the dominant mechanism underlying the control of differential protein expression in trypanosomatids[22], TNKase gene sequence was designed based on trypanosome codon usage. The expression cassette also contains the acid phosphatase signal sequence of *Leishmania mexicana*. Using the new strategy, on day three of growth, the expression level was 810 IU/ml, which was 1.7 times higher than Leishmania strain containing multicopies of t-PA gene[12,17].

The expression level of TNKase was comparable to the truncated form. The expression was higher than full length human rtPA in *L. tarentolae*, 70 IU/ml[10] and than multiple-copy t-PA gene integration transformant (488 IU/ml) [12].

The improved level of expression can contribute to the presence of the T7/TetR system, which is less complicated comparing to mammalian expression systems (Table 2). As the secreted TNKase showed

**Table 2:** Different expression vectors and the level of expression of t-PA in *L. tarentolae*

Protein name	Number of construct transfection	Constructs	Expression level (IU/ml)	Reference
Complete t-PA	Doubly constructs	pFX1.4sat-tPA and pFX1.4hyg-tPA	7	[10]
Complete t-PA	Doubly constructs	pFX1.4sat-2tPA and pFX1.4hyg-2tPA	230-480	[12]
Complete modified t-PA (TNKase)	Single inducible construct	pLxy-I-bleCherry3 (TNKase)	810	This study

similar activity comparing to pharmaceutical dosage form in the market, it can concluded that expressed proteins have similar folding and glycosylation pattern.

The *L. tarentolae* expression system has several advantages in comparison to conventional protein expression systems, which include shorter doubling time (4-6 h), simple manipulation, easy handling[15,23], and homology of the N-glycosylation pattern of *L. tarentolae* with eukaryotic glycosylation structure[8].

In this study, transgenic inducible clones of *L. tarentolae* were developed, which was inducible by tetracycline and could express active TNKase. The functional activity of expressed TNKase was comparable with Activase. The results of our approach in expression and purification of complex proteins, which are among critical and expensive drugs, is promising and with more optimization, we expect an even higher activity and the yield of such valuable drugs.

In conclusion, the results of the present work demonstrate that the addition of T7 promoter/tet operator in *Leishmania* expression vectors can result in higher expression level of target protein. Also, codon usage optimizations of TNKase for expression in *Leishmania* have shown a similar result of increasing the gene dosage in high level expression. We obtained the high level of gene expression, related to the production of secretory heterologous proteins expression using inducible promoter in non-pathogen *Leishmania*. The novel system illustrated here could be proposed as an important alternative to mammalian expression systems which are more expensive and difficult for development.
